# Speeding up GW Calculations to Meet the Challenge of Large Scale Quasiparticle Predictions

**DOI:** 10.1038/srep36849

**Published:** 2016-11-11

**Authors:** Weiwei Gao, Weiyi Xia, Xiang Gao, Peihong Zhang

**Affiliations:** 1 Department of Physics, University at Buffalo, 4 State University of New York, Buffalo, New York 14260, USA; 2 Beijing Computational Science Research Center, Beijing 100084, China; 3 Department of Physics, International Center for Quantum and Molecular Structures, Shanghai University, Shanghai 200444, China

## Abstract

Although the GW approximation is recognized as one of the most accurate theories for predicting materials excited states properties, scaling up conventional GW calculations for large systems remains a major challenge. We present a powerful and simple-to-implement method that can drastically accelerate fully converged GW calculations for large systems, enabling fast and accurate quasiparticle calculations for complex materials systems. We demonstrate the performance of this new method by presenting the results for ZnO and MgO supercells. A speed-up factor of nearly two orders of magnitude is achieved for a system containing 256 atoms (1024 valence electrons) with a negligibly small numerical error of ±0.03 eV. Finally, we discuss the application of our method to the GW calculations for 2D materials.

Accurate predictions of excited states properties are critical for computational screening and design of materials for energy and electronics applications. While density functional theory (DFT)^1^ within the local density approximation (LDA)^2^ or the generalized gradient approximation (GGA)^3^ has been very successful in describing materials ground-state properties, the Kohn-Sham (KS) eigenstates in principle cannot be compared directly with quasiparticle (QP) excitations. The GW approximation^4^,^5^,^6^, systematically derived from the perturbative expansion of electron self-energy, is widely recognized as one of the most accurate methods for predicting excited states properties of materials.

Unfortunately, despite the tremendous success, *accurate* and *efficient* predictions of excited-states properties of complex solid systems remain a major challenge due to complication of the convergence issue^7^,^8^,^9^,^10^,^11^ and the unfavorable scaling of the computational cost (both in terms of the computational time and the storage and memory requirements) with respect to the system size. This is particularly true for systems containing substantially localized electronic states and/or with large unit cells (e.g., nanostructures, complex multinary compounds, surfaces and interfaces, and defects in solids). For example, it has been shown that for simple materials such as ZnO one has to include as many as 3,000 empty states in conventional GW calculations to acheive properly converged results^9^,^11^,^12^,^13^. Although ZnO has probably received the most research attention, the convergence problem of GW calculations is not just limited to this single material. In fact, GW calculations for many systems, including MgO, CuCl, AgBr, and CdO (to name a few), suffer similar convergence problems. Recently, it was shown that about 6,000 bands are need to to converge the GW band gap for monolayer MoS_2_ to within 50 meV^14^, and that for MoSe_2_ is as large as 10,000^15^.

In this work, we present an efficient method that can drastically speed up GW calculations for large systems and is also easy to be implemented within available GW packages. Our method takes advantage of the fact that the the density of states (DOS) of all materials resembles that of the free electron gas at high energies, thus the summation over high-energy conduction in GW calculations can be approximated by a numerical integration on a sparse energy grid. Our method makes fully converged GW calculations for large systems feasible, eliminating the need for performing expensive and tedious convergence tests since one can now afford to include effectively *all* conduction bands in GW calculations at greatly reduced computational costs.

## Results and Discussions

The GW approximation for the electron self-energy Σ(**r**, **r**′; *E*) is


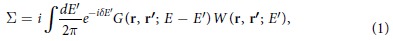


where *δ* = 0^+^, *G* is the one-particle Green’s function, and *W* is the screened Coulomb interaction, which is related to the dielectric function *ε* and the bare Coulomb interaction *V*_*c*_ as *W* = *ε*^−1^*V*_*c*_. In conventional first-principles GW methods^5^,^6^, both the Green’s function and the dielectric functions are constructed using the KS eigenstates, and, in principle, all states in the Hilbert space of the KS Hamiltonian should be included in the summations in calculating *G* and *W*. In practical calculations, truncations are almost always applied. Since the convergence behavior of GW calculations could be very different from materials to materials, it is often difficult to predict *a priori* proper truncation parameters to ensure fully converged GW results. For large systems, it is extremely difficult (if possible at all) to perform fully converged GW calculations within this scheme.

GW calculations involve two computationally expensive summations over conduction bands in calculating the dielectric function *ε* and the Coulomb-hole part of self-energy operator Σ_COH_(*E*)^5^. Within the random phase approximation (RPA), the dielectric function is related to the electron polarizability *χ*^0^ via *ε* = 1 − *V*_*c*_*χ*^0^. The (irreducible) electron polarizability is constructed using KS eigenstates:


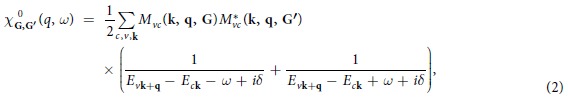


where *M*_*vc*_(**k, q, G**) = 〈*v*, **k** + **q**|*e*^*i*(**q**+**G**)^|*c*, **k**〉, and *v* and *c* index valence and conduction bands. The band summations in the calculations of dielectric function should in principle include *all* conduction (empty) states in the Hilbert space of the KS Hamiltonian. The calculation of the Coulomb-hole (COH)^5^ self-energy also involves a summation over conduction bands^5^,^16^:


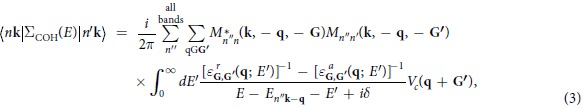


where *ε*^*r*^ (*ε*^*a*^) is the retarded (advanced) dielectric function. The screened exchange (SEX)^5^ part of the self-energy, on the other hand, involves a summation only with occupied states. The convergence of GW results with respect to the number of conduction bands included in the above summations can sometimes be extremely slow, which has become both of a burden and a source of confusion since under-converged results reported by different groups can vary significantly. Our new method *effectively* allows including *all* conduction bands in the above two summations as explained below.

We first use MgO as an example to illustrate the difficulty of scaling up conventional GW calculations for large systems. We then propose our new approach and demonstrate the performance of the newly developed method by showing results for MgO and ZnO. Finally, we discuss the application of our method to GW calculations for 2 dimensional (2D) materials. Due to the slow convergence of the GW results with respect to the *k*-point sampling density, interlayer spacing, and the number of conduction bands included in the calculations, reported quasiparticle band gap of monolayer MoS_2_ ranges from 2.41 to 2.84 eV^14^,^17^, underlining the importance of affordable and efficient GW approaches for reliable predictions of the excited states properties of 2D systems.

### The convergence issue in GW calculations

**Figure 1:**
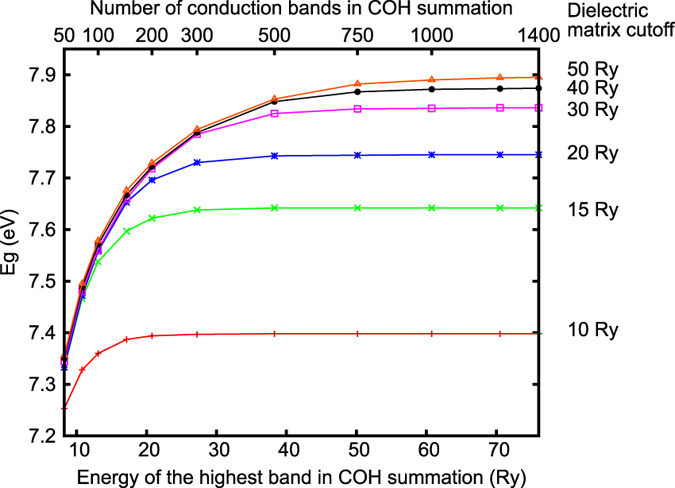
Calculated quasiparticle band gap of MgO as a function of the number of conduction bands *N*_c_, or equivalently, the energy of the highest conduction band, included in the COH self-energy calculations and the kinetic energy cutoff for the dielectric matrices.

To better illustrate the convergence issue in GW calculations, we show in Fig. 1 the convergence behavior of the calculated quasiparticle band gap of MgO (a two-atom primitive cell) as a function of the number of conduction bands included in the COH self energy summation and the kinetic energy cutoff for the dielectric matrices. Depending on the choice of these cutoff parameters, the calculated QP band gap varies from 7.25 to 7.90 eV. Once fully converged, we obtain a QP band gap of about 7.90 eV, in good agreement with the experimental value of 7.78 eV^18^ and a previous calculation^11^. As it is shown in Fig. 1, a high kinetic cutoff (|**G**_cut_|^2^/2 ~ 40 Ry) for the dielectric matrices *ε*_**G**,**G′**_(**q**, *ω*) and a large number of conduction bands (*N*_*c*_ ~ 800) in the COH summation are required. If a small kinetic energy cutoff for the dielectric matrices is used, the band gap converges quickly but prematurely with respect to the number of band included in the summation.

With today’s massively parallel computers, fully converged GW calculations for small systems such as 2-atom MgO can be done easily. The problem, however, quickly becomes intractable when one attempts to scale up the calculations for large systems containing hundreds of atoms because the number of conduction bands required in the COH and dielectric function calculations scales linearly with the system size (i.e., number of atoms). To put this problem in perspectives, suppose now we would like to carry out GW calculations for a 128-atom MgO supercell containing a defect (e.g., an F-center), the required number of bands would increase by 64 (i.e., 128/2) times. Not only it is extremely difficult to generate so many wave functions, storing these wave functions would also pose a serious challenge, not to mention performing the band summations in the GW calculations.

Considering the importance of predicting materials excited states properties, it is not surprising that there has been much work on improving the efficiency of the GW formalism for large systems beyond simple crystals. One direction is to introduce better (optimized) bases for representing the dielectric matrix and/or the self-energy operator beyond plane waves and KS eigen states^19^,^20^,^21^. The second direction is to reduce the number of conduction bands *N*_*c*_ by replacing the summation over high-energy states with computationally tractable terms^7^,^8^,^11^,^22^,^23^, or completely removing the necessity of summing over unoccupied states^24^,^25^,^26^. Other efforts include applications of the Lanczos algorithm for calculating the dielectric functions^27^,^28^, the development of the stochastic GW approach^29^, and the separation of the dynamical and nonlocal correlations^30^ in dynamical mean field theory (DMFT) calculations which may also be applied to GW calculations. These efforts underline the urgency of developing efficient GW methods to meet the challenge of fast and accurate predictions of the excited states properties of large systems such as nanostructures, defects in solids, and surfaces and interfaces. Some of these approaches, however, may require substantial modifications (or completely rewriting) existing GW codes, and the study of the convergence behavior of these methods is still in the early stage.

### Replacing the band-by-band summation with an energy integration in GW calculations

**Figure 2:**
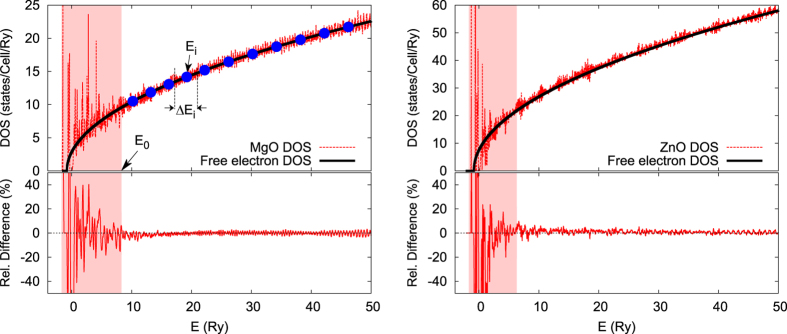
Comparison between the DOS of MgO (left panel) and ZnO (right panel) calculated within the LDA (red) and that of free electron gas (black). The DOS for the free electron gas is shifted with respect to the averaged exchange-correlation potential. The relative difference between the LDA DOS and that of free-electron gas DOS as a function of energy is also shown to gauge the quality of the approximation.

Our approach to speeding up the calculation is inspired by the observation that the electron DOS of all materials can be divided into two regions: a low energy region in which the behavior of electronic states is strongly affected by the crystal potential (thus materials dependent) and a high energy region in which kinetic energy dominates and the DOS scales as 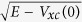 as that for free electron systems, where *V*_*xc*_(0) is the averaged exchange-correlation potential of the material. Figure 2 compares the DOS of MgO and ZnO calculated within the LDA and that of the free-electron gas scaled with the volume of the respective unit cells. The DOS below certain energy (*E*_0_ shown in left panel of Fig. 2) is what distinguishes one material from another. Above *E*_0_, the DOS can be well-approximated by that of free-electron gas.

This observation leads us to propose that the band summation in the GW calculations be divided into two regions: a low energy region (shaded area in Fig. 2) in which the band summation is carried out explicitly and a high-energy region in which the band summation is replaced (approximated) by an energy integration. For example, the static polarizability *χ*^0^ is rearranged in the following form:


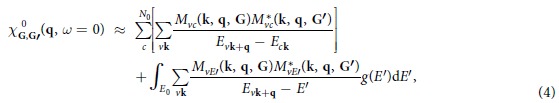


where 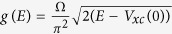 is the approximated DOS for the system with a cell volume Ω, *E*_0_ corresponds to the energy of band *N*_0_, and *M*_*vE*_(**k, q, G**) are the matrix elements between the valance band *v* and the conduction band at (or near) the energy *E*. In practical calculations, we find that the number of bands *N*_0_ can be limited to less than 10 per atom for most systems. The auxiliary integral part can be carried out using simple numerical integration techniques by sampling the electronic states on an energy grid as illustrate in left panel of Fig. 2:


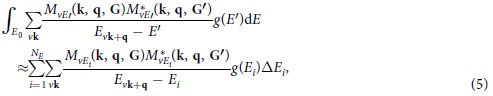


where *N*_*E*_ is the number of sampling points used in the numerical integration as shown schematically in the left panel of Fig. 2 with large blue dots. The same approach is applicable to the calculation of the COH self-energy. The integration can be carried on a uniform or nonuniform energy grid, and a typical energy step Δ*E* can range from 1 to 5 eV. Higher order numerical integration techniques may also be applied.

### Demonstration of the accuracy and efficiency of the new method

**Figure 3:**
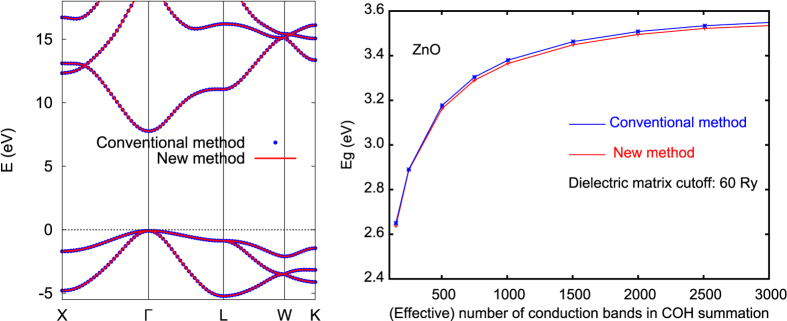
Comparison of GW results calculated using conventional method (blue) and new method (red). Left: GW band structure of MgO; right: GW band gap of ZnO as a function of number of bands included in COH summation.

We now examine the accuracy of this new method. We first compare the GW band structure of MgO (2-atom primitive cell) calculated using the conventional approach and our new method as shown in left panel of Fig. 3. The difference is unnoticable at this scale. We have also tested the result for ZnO. The right panel of Fig. 3 shows the calculated GW band gap of ZnO as a function of number bands included in the COH self-energy summation (and in the dielectric matrix calculations). The difference between the results calculated using the two methods is again negligibly small (~0.02 eV). For the calculation using our new method, the number of integration points plus the number of bands within the energy window *E*_0_ is about 350, to be compared with 3,000 bands in conventional GW calculations.

We then test the stability and performance of our method using a reasonably small (but not too small) system, an MgO supercell containing 16 atoms, as an example. For this system, we choose *N*_0_ to be 128. In other words, 8 conduction bands per atom are included in the explicit band-by-band summations in the calculation of the dielectric matrices and the COH self-energy. The auxiliary integral part is calculated on a uniform energy grid with a step Δ*E* ranging from 1.5 to 4.0 eV, and the integration is carried out up to an energy which is equivalent to including 8,000 (or 1,000 per 2-atom cell) conduction bands. The kinetic energy cutoff for the dielectric matrix is set at 40 Ry.

For such a small system, we can compare directly the results calculated using the new method and the conventional band-by-band summation approach, as shown in Table 1. As the energy integration step varies from 1.5 to 4 eV (while keeping *N*_0_ fixed at 128), the number of conduction bands plus the number of the energy integration points decreases from 895 to 320, to be compared with 8,000 required in conventional calculations to achieve the same level of convergence, representing a speed-up factor of 9 to 25. It is rather encouraging that the results calculated using this new method are insensitive to the energy integration step Δ*E*, showing the stability of the this method. The GW band gap calculated with the conventional method is 7.86 eV, and that obtained from the new method only deviates by a negligible small amount of ±0.02 eV.

Our method reduces the computational time of fully converged GW calculations for a 16-atom MgO supercell from about 5 hours to 15 minutes using 64 computing cores (8 Intel Xeon E5-2650 processors). We would like to mention that within this new approach, one only needs to calculate (and store) the wave functions of the *N*_0_ low energy states plus selected high-energy conduction states at or near predefined energy grid points. The folded-spectrum method developed by Wang and Zunger^31^ and an alternative approach proposed by Tackett and Ventra^32^ are ideal for this purpose. Using these methods, one can efficiently calculate KS eigenfunctions at or near specified energy grid points without the need to calculate all KS states. Therefore, the speed-up factor shown in Table 1 also indicates the reduction in the required memory and disk space associated with storing the wave functions in GW calculations.

The most interesting and important aspect of our method is that the speed-up factor actually increases with increasing system size, enabling fully converged GW calculations for large systems containing hundreds of atoms. This is because with a given integration step size Δ*E*_*i*_, the weight of a single integration point, *g*(*E*_*i*_)Δ*E*_*i*_ in Eq.5, which measures effectively the number of states contributing to the summation represented by a single state at *E*_*i*_, scales linearly with the system size. To demonstrate that our method can indeed be scaled up for large systems, we have carried out GW calculations for MgO systems containing 2 to 256 atoms.

Table 2 shows the performance of our method, both in terms of accuracy and the speed-up factor. A speed up factor of over 80 times is achieved for the largest system containing 256 atoms (1,024 valence electrons), and the numerical integration error, measured by the calculated band gap, is only ±0.03 eV for all systems. Thus the greater speed-up for larger systems does not compromise the accuracy of this method, and there is still room for improvement (for example, with improved numerical integration techniques). We would like to mention that such *fully converged* GW calculations for large oxide systems containing over 200 atoms would be extremely difficult using the conventional approach due to the vast amount of computational resources required (both in terms of the computational time and the memory and storage requirements).

### Applications to 2D materials

GW calculations for 2D materials pose additional challenges due to the analytical behavior of the 2D electronic screening, which is different from 3 dimensional (3D) systems, and the need to remove interactions between period images^33^,^34^,^35^,^36^. The rapid change in the 2D dielectric function as 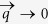 requires rather dense *k*-point sampling even with clever integration techniques^33^,^36^; the spurious interlayer interaction can be reduced with increasing interlayer distance and with the use of truncated Coulomb potential^33^,^34^. Unfortunately, even with these creative methods, fully converged GW calculations for 2D materials remain a serious challenge since the number of conduction bands required in the calculation scales linearly with cell volume. For example, Qiu *et al*. concluded that at least 6,000 bands are needed for properly converged GW results of monolayer MoS_2_.

**Figure 4:**
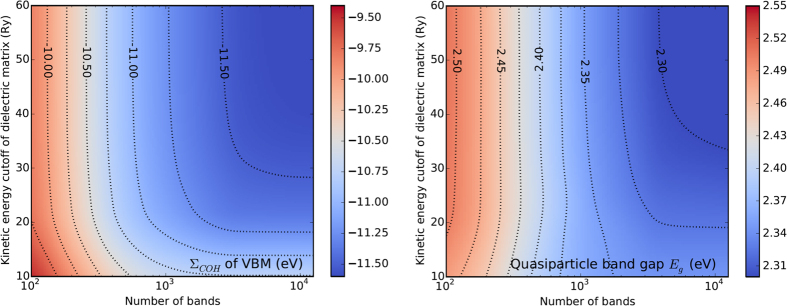
Convergence behavior of the COH self-energy Σ_*COH*_ (left panel) for the top of the valence band and the quasiparticle band gap *E*_*g*_ (right panel) of MoSe_2_ as a function of the number of conduction bands and the kinetic energy cutoff of the dielectric function. The GW calculations are carried out using a 21 × 21 × 1 k-grid.

Our method can be directly applied to 2D materials in the same way as 3D bulk materials. Using this new method, we thoroughly studied the convergence behavior of monolayer MoSe_2_. The left panel of Fig. 4 shows the convergence behavior of the calculated COH self-energy for the top of the valence band of MoSe_2_ as a function of the number of conduction bands and the kinetic energy cutoff of the dielectric function. Depending on the the choices of these parameters, the calculated Σ_*COH*_ varies from less than −11.5 eV to about −9.5 eV. The right panel of Fig. 4 shows a similar convergence behavior of calculated quasiparticle band gap *E*_*g*_ of MoSe_2_. The variation in band gap, although not as large as that in the COH enegy due to cancellation of errors between valence and conduction bands, is still significant. These results again demonstrate the importance of the band convergence issue in GW calculations, and often seemingly conflicting GW results can be traced to the convergence issue.

**Figure 5:**
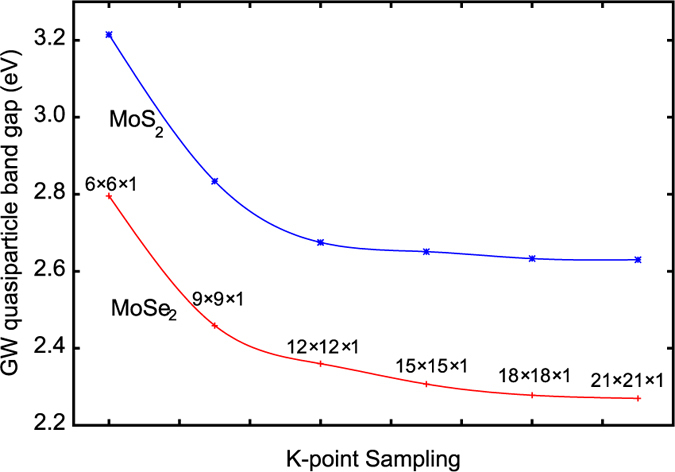
Convergence behavior of the calculated GW quasiparticle band gap *E*_*g*_ of MoSe_2_ and MoS_2_ as a function of the k-point sampling density.

We mention that GW calculations for 2D materials usually converge much slower with respect to k-point sampling than 3D systems^33^,^35^. if one employs truncated Coulomb potentials, which is usually required to avoid spurious influences from periodic images. As shown in Fig. 5, for monolayer MoS_2_ and MoSe_2_, one has to increase the k-grid sampling to at least 18 × 18 × 1 in order to converge the quasiparticle band gap. In contrast, 6 × 6 × 6 k-grids are usually sufficient for typical bulk materials with small unit cells containing a few atoms. Our calculations make use of the truncation technique proposed by Ismail-Beigi^33^. This issue significantly increases the computational costs for GW calculations for 2D systems and makes the convergence test even more trickier and difficult due to possible cancellations of errors arising from various cut-off parameters. Our new method enables including effectively *all* conduction bands in the GW calculations with greatly reduced computational costs (by one to two orders of magnitude) compared with conventional methods. Using this new method, we have calculated GW band gap of free-standing monolayer MoSe_2_ to be 2.28 eV, consistent with previously reported value 2.26 eV^15^, and the band gap of monolayer MoS_2_ to be 2.64 eV, which is also in good agreement with a previously calculation^37^. These previous results were obtained with highly converged (but computationally expensive) conventional GW calculations.

## Conclusion

In conclusion, we present an efficient integration method that can drastically speed up fully converged GW quasiparticle calculations for large systems. Our method takes advantage of the fact that the DOS of all materials resembles that of the free electron gas at high energies, thus the summation over high-energy conduction bands in both the dielectric function and self-energy calculations within the GW approximation can be well approximated by a numerical integration on a sparse energy grid. Using this new method, we can now carry out fully converged GW calculations for large systems without the need to perform tedious convergence tests. We have demonstrated a nearly two orders of magnitude speed-up of GW calculations for a 256-atom MgO model system while achieving a numerical accuracy of ±0.03 eV for the predicted band gap. Although we have presented results calculated within the so-called G^0^W^0^ approximation and using the HL-GPP model for the dielectric function, we believe that our approach can be applied to various levels of self-consistent GW calculations and/or GW calculations without the use of plasmon-pole models. Our new method also works equally well for 2D systems. Our approach is conceptually simple and is very easy to be implemented in well-developed GW packages.

## Methods

All GW results presented in this work are calculated within the G^0^W^0^ approach using a modified version of the BerkeleyGW^16^ package. The Hybersten-Louie generalized plasmon-pole (HL-GPP) model^5^ is used to extend the static dielectric function to finite frequencies. The new method presented in this work, however, can be applied to other GW implementations including various self-consistent GW approaches. The DFT calculations within the LDA are carried out using the PARATEC^38^ code. Norm-conserving Troullier-Martin pseudopotentials^39^ are used. The kinetic energy cutoff for the plane-wave expansion of the KS wave functions is set at 80 Ry for MgO, 300 Ry for ZnO, and 125 Ry for monolayer MoSe_2_ and MoS_2_. Experimental crystal structures are used for all calculations. For monolayer MoSe_2_ and MoS_2_, the interlayer distance is kept at 25 Å. All semicore electrons (4*s* and 4*p* for Mo and 3*s* and 3*p* for Zn) are included in the calculations. The GW quasiparticle band structures for MgO 2-atom cell are obtained using a Wannier interpolation technique^40^ based on results calculated on a 8 × 8 × 8 *k*-grid.

## Additional Information

**How to cite this article**: Gao, W. *et al*. Speeding up GW Calculations to Meet the Challenge of Large Scale Quasiparticle Predictions. *Sci. Rep.* **6**, 36849; doi: 10.1038/srep36849 (2016).

**Publisher’s note**: Springer Nature remains neutral with regard to jurisdictional claims in published maps and institutional affiliations.

## Figures and Tables

**Table 1:** Calculated GW band gap (in eV) of a 16-atom MgO cell: accuracy and speed-up factor of the new method.

Δ*E* (eV)	New method		Conventional method		Speed-up factor	Δ*E*_*g*_
	*N*_0_ + *N*_*E*_	*E*_*g*_	*N*_*c*_	*E*_*g*_		
1.5	895	7.84	8,000	7.86	8.9	−0.02
2.0	640	7.86			12.5	0.00
2.5	510	7.88			15.7	+0.02
3.0	435	7.88			18.4	+0.02
3.5	385	7.86			20.8	0.00
4.0	320	7.84			25.0	−0.02

**Table 2:** Performance of the new method with increasing system size.

# of atoms	New method		Conventional method		Speed-up factor	Δ*E*_*g*_
	*N*_0_ + *N*_*E*_	*E*_*g*_	*N*_*c*_	*E*_*g*_		
2	170	7.86	1,000	7.86	5.9	0.00
16	320	7.84	8,000		25.0	−0.02
64	920	7.89	32,000		34.8	+0.03
128	1060	7.83	64,000		60.4	−0.03
256	1580	7.86	128,000		81.0	0.00
